# Early Nutritional Programing: Unlocking the Potential of Fish for Sustainable Aquaculture

**DOI:** 10.1155/anu/3380126

**Published:** 2026-01-15

**Authors:** Shivendra Kumar, Aditi Banik, Maneesh Kumar Dubey, Prem Prakash Srivastava, Zsuzsanna J. Sandor

**Affiliations:** ^1^ Department of Aquaculture, College of Fisheries (Dr. Rajendra Prasada Central Agricultural University), Dholi, Muzaffarpur, 843121, Bihar, India; ^2^ Research Centre of Aquaculture and Fisheries, Hungarian University of Agricultural and Life Sciences, Anna liget. u. 35, 5540, Szarvas, Hungary, uni-mate.hu; ^3^ College of Fisheries (Dr. Rajendra Prasada Central Agricultural University), Dholi, Muzaffarpur, 843121, Bihar, India

**Keywords:** epigenetics, larval fish, maternal nutrition, metabolic programing

## Abstract

Nutritional programing, which explores the link between early nutritional conditions and their long‐term effects on animals, is a developing field within fish biology. Suboptimal nutritional status during early life is strongly associated with a higher risk of metabolic consequences later in life, including permanent growth retardation, impaired neural development, and disruption of important metabolic pathways. This association has been demonstrated by epidemiological evidence and subsequent studies conducted using fish models. It appears that fish raised on endogenous (maternally derived) and exogenous (larval feeding) diets from an early age may have comparable developmental and metabolic programing effects. Nutritional programing in fish has been shown to have an impact on survival, growth, cognitive advancement, and metabolism of nutrients. The influence of these programing effects may be facilitated by changes in metabolic pathways and the epigenetic regulation of gene expression during a critical window when bodies demonstrate great developmental plasticity. Thus, one strategy to encourage sustainable feeding practices in aquaculture may be nutritional programing. Furthermore, there is a significant death rate in the early stages of life within this critical window. This implies that the dynamics of wild populations may have quantifiable repercussions as a result of programing impacts. Numerous significant concerns remain unresolved due to the diversity of fish and the vast range of metabolic effects of programing. This review summarizes findings from fish models and emphasizes the existing knowledge gaps as well as key research priorities in the field of nutritional programing in fish.

## 1. Introduction

Nutritional programing in animals refers to the concept that early‐life nutrition can have long‐term effects on an animal’s growth, metabolism, reproduction, and overall health. This concept is based on the developmental origins of health and disease (DOHaD) hypothesis, which suggests that nutritional and environmental factors during critical periods of development can induce physiological changes that persist throughout life [1]. In livestock and companion animals, nutritional programing has gained increasing attention as a tool for improving productivity, disease resistance, and feed efficiency [2].

Early‐life nutritional interventions, such as prenatal and neonatal feeding strategies, can influence gene expression, organ development, and metabolic pathways, ultimately shaping an animal’s performance and health status. For example, in pigs, maternal nutrition during gestation can affect offspring birth weight and postnatal growth rates [3]. Similarly, in ruminants, early‐life nutrition can alter rumen development and nutrient utilization efficiency [4]. Understanding these mechanisms allows for the development of precise feeding programs that enhance animal welfare and production sustainability. As nutritional programing continues to evolve, researchers explore how specific nutrients, bioactive compounds, and feeding regimens contribute to lifelong physiological changes. This knowledge has broad implications for animal agriculture, companion animal nutrition, and even conservation efforts. By leveraging nutritional programing, producers and pet owners can optimize animal health and productivity while reducing environmental impacts.

In aquaculture, nutritional programing has gained significant interest as a tool for optimizing feed efficiency, improving growth rates, and enhancing disease resistance, thereby contributing to the sustainability and profitability of fish farming [5]. Early‐life nutrition, particularly during embryonic, larval, and juvenile stages, plays a crucial role in shaping fish development and metabolic pathways [6]. Studies have shown that specific dietary interventions during these critical windows can lead to persistent modifications in nutrient utilization, digestive enzyme activity, and immune responses [7]. For instance, dietary programing with specific amino acid profiles or lipid sources in larval fish has been found to enhance protein deposition and growth performance in later stages [8]. Similarly, maternal nutrition in broodstock can influence offspring quality by altering yolk composition, which in turn affects early‐stage development and survival rates [9]. One of the key mechanisms underlying nutritional programing in fish is epigenetic regulation, including DNA methylation, histone modifications, and microRNA expression, which can influence gene expression without altering the genetic code [10]. These molecular changes enable fish to adapt to specific dietary conditions, potentially leading to improved resilience against environmental stressors such as fluctuating water temperatures and pathogen exposure [11]. Furthermore, nutritional programing can be leveraged to reduce reliance on traditional fishmeal‐based diets by conditioning fish to efficiently utilize alternative protein and lipid sources, thus promoting sustainable aquafeed formulations [12].

As the aquaculture industry seeks to improve production efficiency while minimizing environmental impacts, nutritional programing offers a promising strategy to enhance fish performance through targeted early‐life feeding interventions. Understanding the biological mechanisms and long‐term effects of nutritional programing will be crucial for developing precise feeding protocols tailored to different fish species and production systems. Future research should focus on elucidating species‐specific responses to early dietary manipulations, optimizing feed formulations, and integrating omics‐based approaches to further unravel the complexities of nutritional programing in fish.

## 2. Concept of Nutritional Programing in Animals and Fish

**Figure 1:**
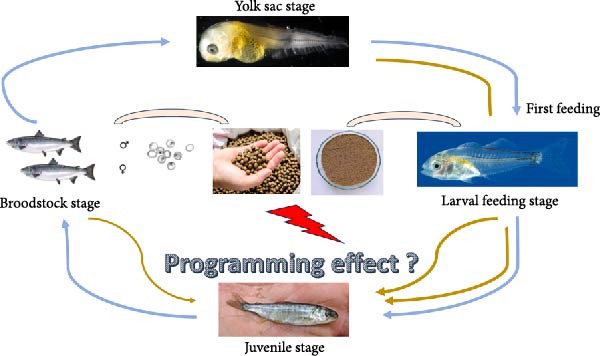
Possible feeding stimuli at a critical stage for nutritional programing in fish.

This concept is rooted in the DOHaD hypothesis, which suggests that environmental influences, including nutrition during early developmental stages, can induce permanent physiological and metabolic changes [1]. It has been extensively researched in mammals to comprehend the implications in later stages of disrupted nutrition in the intrauterine or postnatal phases [13–16]. Throughout development, the phenomenon of plasticity enables the generation of diverse phenotypes from identical genotypes [17], particularly notable in specific species [18]. There are hints that the mechanism of developmental plasticity leverages nutritional signals at early stages to prepare specific phenotypes for suitable adaptation to anticipated future nutritional conditions [19]. The early developmental and neo‐natal periods, characterized by the formation of various tissues and organs, are distinguished by their heightened molecular‐level plasticity [14, 17]. These phases are identified as optimal developmental windows during which programing could be most effective. Furthermore, potential biological mechanisms behind “nutritional imprinting,” which involves recollection of a previous nutritional occurrence in later life in mammalian craniates, encompass adaptive alterations in gene expression and/or cell‐mediated traits (associated with epigenetic mechanisms), nutrient‐responsive signaling pathways, and adaptive clonal selection that may be passed on to future progeny [20, 21]. Hence, the stages for implementing a nutritional stimulus during phases of sensitive developmental flexibility are limited to (i) maternal nutrition, through the transfer of nutrients to yolk reserves and/or alterations in gamete epigenetics, and (ii) the initiation of external feeding (Figure 1). This is referred to as Programing window or critical window, indicating the phase of life during which nutritional interventions were implemented to elicit effects on one or more physiological functions in animals, including fish. Nutritional programing in fish operates through multiple mechanisms that enable fish to adapt to specific dietary and environmental conditions. These mechanisms may be either through epigenetic modification (DNA methylation, histone modification, microRNA regulation), metabolic imprinting (amino acid programing, lipid metabolism programing, glucose utilization), organ developmental adaptation (structural and functional development in key vital organs like gut, lung, pancreas, muscle tissue, etc.) or nutrient sensing and hormonal regulation (leptin and ghrelin modulation, insulin like growth factor‐1).

## 3. Studies on Fish: Role of Maternal‐Derived Nutritional Cues

Maternal‐derived nutritional cues play a fundamental role in fish development, influencing offspring survival, growth, metabolism, and overall fitness. These cues, transmitted from the mother to the developing embryos via yolk reserves, hormones, and other biochemical compounds, contribute to the phenomenon of nutritional programing (Table 1), which induces long‐term physiological and metabolic adaptations in offspring [9, 32]. Recent research highlights how maternal nutrition, environmental conditions, and epigenetic modifications shape early development, affecting larval success and later‐life performance in aquaculture species [10, 33]. Understanding the role of maternal‐derived nutrients is crucial for optimizing broodstock diets, enhancing seed quality, and improving aquaculture productivity.

**Table 1:** Summary of the fish nutritional programing studies evidenced with context to maternally derived nutritional stimuli.

Species	Maternal diet/stimulus	Observed effects in progeny	Mechanism involved	References
Zebrafish	High‐carbohydrate diet	Enhanced hepatic glycolytic gene expression, improved glucose tolerance	Epigenetic regulation (DNA methylation)	[7]
Rainbow trout	High‐plant protein diet	Offspring exhibited improved growth on plant‐based diets	Nutritional imprinting	[22]
Nile tilapia	High‐fat maternal diet	Increased lipid storage, altered growth trajectory	Modulation of lipid metabolism genes	[23]
European sea bass	High‐carbohydrate maternal diet	Modified offspring hepatic glucose metabolism and enzyme activities	mRNA and epigenetic modulation	[24]
Gilthead seabream	Plant‐ingredient enriched maternal diet	Enhanced utilization of plant proteins, improved gut morphology	Gut microbiota modulation, transcriptional shifts	[25]
Atlantic salmon	n‐3 PUFA‐deficient maternal diet	Altered offspring fatty acid profiles, reduced growth	Lipid metabolic imprinting	[26]
Common carp	Carbohydrate‐rich maternal diet	Increased expression of gluconeogenic enzymes in offspring	Metabolic flexibility via gene expression	[27]
Senegalese sole	Maternal PUFA and vitamin supplementation	Growth, development (deformity), lipid metabolism	Lipid metabolic imprinting	[28]
Yellow catfish (*Tachysurus fulvidraco*)	High‐carbohydrate diet	Improved hatching parameters, metabolome, Upregulated glucose metabolism related gene expressions, and global DNA methylation	Metabolic imprinting and genetic modulation	[29]
Common carp	Plant‐based diet	Improved growth performance and nutrient utilization	Nutritional imprinting	[30]
Nile tilapia	Purified nucleotide	Improved health, growth, and the resistance face to transport and challenge against *Aeromonas hydrophila* in juveniles	Nutritional imprinting	[31]

### 3.1. Maternal Nutritional Transfer and Its Effects on Offspring

Maternal provisioning of nutrients to offspring occurs primarily through the yolk, which supplies essential macronutrients, including proteins, lipids, and carbohydrates, as well as vitamins, minerals, and bioactive compounds. The composition of the yolk is directly influenced by the maternal diet, which in turn determines larval viability and growth performance [5, 34]. Several studies have examined how variations in maternal nutrition affect yolk composition and subsequent offspring development.

#### 3.1.1. Influence on Egg Quality and Yolk Composition

The nutrients provided by the mother directly contribute to the yolk reserves of developing embryos, determining their initial energy stores and biochemical composition. High‐quality eggs are characterized by optimal levels of proteins, lipids, essential fatty acids, vitamins, and minerals, which are crucial for embryogenesis and larval survival [28]. For example, in Senegalese sole (*Solea senegalensis*), broodstock fed with diets enriched in polyunsaturated fatty acids (PUFAs) produced eggs with significantly higher levels of eicosapentaenoic acid (EPA) and docosahexaenoic acid (DHA), resulting in improved larval development and reduced deformities [28]. Likewise, maternal supplementation with carotenoids and vitamins has been shown to enhance egg quality. In contrast, deficiencies in maternal micronutrients, such as vitamin B_12_, folate, and methionine, resulted in impaired yolk utilization and delayed embryonic development in zebrafish (*Danio rerio*) [35, 36].

#### 3.1.2. Metabolic Programing and Growth Performance

Maternal nutrition plays a crucial role in metabolic programing, which influences the offspring’s ability to utilize nutrients efficiently. This programing occurs through modifications in gene expression, enzyme activity, and hormonal regulation during early development. For instance, a study on gilthead seabream (*Sparus aurata*) demonstrated that replacing fish oil (FO) with plant‐based oils in the maternal diet altered the metabolic pathways of offspring, improving their ability to utilize alternative lipid sources in later life [37]. Similarly, Fuiman and Perez [38] showed that red drum (*Sciaenops ocellatus*) larvae originating from eggs enriched with DHA exhibited higher growth rates, better swimming performance, and enhanced survival compared to those from a standard maternal diet. Additionally, early‐life exposure to specific dietary components can shape long‐term metabolic traits. In rainbow trout, brief exposure to a plant‐based diet during the fry stage enhanced feed conversion efficiency and growth performance when the same diet was reintroduced later in life, highlighting the role of early dietary programing [22].

#### 3.1.3. Effects on Immune Function and Disease Resistance

Maternal nutrition also affects immune system development in offspring, influencing their ability to resist infections and environmental stressors. This is particularly important in aquaculture, where disease outbreaks can significantly impact production. Findings have shown that broodstock diets enriched with immunostimulants, such as nucleotides, probiotics, and specific fatty acids, enhance larval immunity [39]. Similarly, larvae from gilthead seabream broodstock fed with essential fatty acid‐enriched diets exhibited higher survival rates when challenged with pathogens, suggesting improved immune function [5]. Furthermore, deficiencies in maternal micronutrients can compromise immune responses in offspring. Zinc and selenium deficiencies in broodstock diets have been linked to impaired antioxidant defenses in larvae, increasing susceptibility to oxidative stress and disease [40].

#### 3.1.4. Effects on Reproductive Success and Offspring Quality

Maternal diet also has implications for reproductive success and offspring fitness, influencing spawning success, egg viability, and larval quality. In species that rely on body reserves for yolk formation, such as some marine fish, prolonged maternal malnutrition can lead to reduced fecundity and poor‐quality offspring [40]. In European seabass (*Dicentrarchus labrax*), maternal diets rich in omega‐3 fatty acids improved reproductive performance, increased egg fertilization rates, and enhanced larval robustness [41]. Conversely, maternal malnutrition, particularly deficiencies in essential fatty acids and vitamins, has been associated with reduced egg viability and increased larval deformities in multiple species [42, 43]. Moreover, studies suggest that maternal nutrition can influence sperm quality in fish. Research on gilthead seabream showed that broodstock diets supplemented with specific micronutrients improved sperm motility and fertilization success [44]. These findings highlight the importance of optimizing maternal and paternal diets to enhance reproductive efficiency in aquaculture.

### 3.2. Phenomena Underlying the Effects of Maternal Nutrition on Offspring Development

The effects of maternal nutrition on offspring development are driven by several interconnected biological phenomena. These include nutritional programing, maternal effect theory, metabolic imprinting, and developmental plasticity. These mechanisms regulate early embryonic development, metabolic efficiency, immune function, and long‐term adaptability in fish species.

#### 3.2.1. Nutritional Programing and Maternal Effect Theory

Nutritional programing, often referred to as the “nutritional imprinting hypothesis,” suggests that maternal nutrition during embryonic and early larval stages affects metabolic pathways and influences growth, survival, and fitness later in life [45]. Nutrients provided in the yolk influence the offspring’s ability to utilize specific dietary components efficiently. Alterations in maternal diet composition can lead to metabolic reprograming, where the offspring develops specific enzyme activities, hormone levels, and digestive efficiencies tailored to available nutrient sources. For instance, rainbow trout (*Oncorhynchus mykiss*) fry briefly exposed to a plant‐based diet exhibited improved adaptation to plant proteins later in life, demonstrating the long‐term metabolic effects of early‐life dietary exposure [22].

Maternal effects describe how the phenotype of an offspring is influenced by the maternal environment rather than direct genetic inheritance. This is a nongenetic form of inheritance in which maternal provisioning of nutrients, hormones, and biochemical factors determines offspring traits [46]. Maternal provisioning through egg yolk composition regulates offspring development and survival. Maternal diet influences the presence of bioactive compounds such as hormones (e.g., cortisol and thyroid hormones) and fatty acids (e.g., DHA and EPA), which control developmental processes as observed in red drum (*S. ocellatus*), where maternal supplementation of DHA led to increased swimming performance and improved survival, emphasizing the role of maternal effects in shaping offspring fitness [38].

As a consequence, the development of specific enzyme activities, hormone levels, and digestive efficiencies in fish offspring is primarily driven by nutritional programing and metabolic imprinting. These mechanisms ensure that larvae can efficiently utilize nutrients inherited from maternal yolk reserves and those encountered in their external environment post‐hatching, optimizing survival, growth, and overall fitness [5, 38]. During embryonic development, maternal nutrition plays a crucial role in shaping metabolic pathways. Nutrients supplied through the yolk sac serve as both an energy source and biochemical signals that influence gene expression. Specific macronutrients, such as proteins, lipids, and carbohydrates, regulate the activity of digestive enzymes. For instance, eggs enriched with high levels of PUFAs result in larvae with increased lipase activity, enabling efficient fat digestion [28]. Similarly, maternal protein levels determine the expression of proteolytic enzymes such as trypsin and pepsin, essential for breaking down dietary proteins [34]. This preconditioning ensures that larvae possess an optimal enzymatic profile suited to the type of nutrients they will encounter posthatching.

#### 3.2.2. Metabolic Imprinting

Metabolic imprinting is a process where early‐life nutritional conditions establish lasting metabolic traits. It is a subset of nutritional programing that affects how nutrients are processed, stored, and utilized throughout life [1]. Maternal diet influences key metabolic enzymes and pathways in offspring, affecting lipid metabolism, energy storage, and protein utilization. Exposure to specific macronutrient compositions in early development alters the activity of metabolic regulators such as peroxisome proliferator‐activated receptors (PPARs) and insulin‐like growth factors (IGFs), which influence growth and metabolic efficiency [1]. In gilthead seabream (*S. aurata*), offspring from broodstock fed plant‐based diets showed improved utilization of alternative feed sources, demonstrating metabolic adaptation to maternal diet [37].

Metabolic imprinting further reinforces these early adaptations by permanently influencing metabolic pathways. This phenomenon occurs when specific nutrients encountered during embryonic and early larval stages induce long‐term changes in enzyme regulation and hormone levels. For instance, larvae that experience high maternal carbohydrate provisioning develop an upregulated amylase system, allowing them to efficiently digest plant‐based diets later in life [22]. Conversely, offspring from fish that primarily consume lipid‐rich diets show an enhanced capacity for fat oxidation, ensuring energy efficiency when consuming lipid‐heavy feed [5]. These metabolic modifications arise due to alterations in gene transcription, where certain metabolic enzymes are upregulated while others are suppressed based on early dietary exposure [37].

#### 3.2.3. Developmental Plasticity

Developmental plasticity refers to an organism’s ability to modify its phenotype in response to environmental conditions experienced during early development [47]. Early exposure to specific dietary and environmental conditions allows fish larvae to develop physiological traits that match future environments. This plasticity is crucial in aquaculture, where optimizing early nutrition can improve growth rates, feed efficiency, and stress resilience, as reported in brown trout (*Salmo trutta*) larvae, where mothers fed salt‐enriched diets exhibited changes in osmoregulatory gene expression, improving their ability to transition to seawater environments [48].

Hormonal regulation plays a complementary role in ensuring metabolic adaptation aligns with nutrient availability. Thyroid hormones such as triiodothyronine (T3) and thyroxine (T4) influence metabolic rate, digestive efficiency, and developmental timing. Higher maternal provision of iodine, an essential component of thyroid hormones, leads to increased T3 and T4 levels in larvae, accelerating metabolism and improving nutrient assimilation [49]. IGF‐1 regulates muscle development and growth, and its expression is directly influenced by maternal protein intake. In fish species where mothers consume protein‐rich diets, offspring exhibit higher IGF‐1 levels, leading to improved protein synthesis and faster growth [50]. Additionally, the hormone leptin, which governs appetite regulation, is influenced by maternal lipid levels, ensuring that larvae adjust their feeding behavior in response to the energy density of their environment [51].

### 3.3. Implications for Sustainable Aquaculture

The epigenetic effects of maternal diet provide new insights into optimizing broodstock feeding strategies for aquaculture. Many economically significant fish species rely on fishmeal and FO‐based diets, which pose sustainability challenges. While alternative plant‐based diets have been explored, their impact on growth, immune function, and nutritional quality remains a concern [41, 42]. However, some species exhibit a degree of metabolic plasticity that allows them to adapt to plant‐based diets through early‐life nutritional programing. For instance, Geurden et al. [22] found that rainbow trout (*O. mykiss*) fry briefly exposed to a plant‐based diet exhibited greater acceptance and utilization of plant proteins later in life. Similarly, gilthead seabream (*S. aurata*) broodstock fed diets replacing FO with linseed oil produced juveniles that efficiently utilized alternative feed sources [5]. These findings highlight the potential for early nutritional programing to improve the sustainability of aquaculture by reducing reliance on marine‐derived feed ingredients. Furthermore, research into broodstock nutrition has demonstrated its impact on reproductive success and sperm quality. Studies suggest that micronutrients such as folate, methionine, and specific polyphenols can influence sperm quality and offspring performance by altering epigenetic marks [50, 51].

Nutritional cues provided during embryonic and early larval stages shape physiological traits via metabolic and epigenetic pathways, with potential applications in sustainable aquaculture. Further research is needed to refine broodstock feeding strategies and understand species‐specific responses to maternal dietary interventions. By leveraging epigenetics and early‐life nutritional programing, aquaculture can optimize productivity while reducing reliance on fish‐derived feed sources, enhancing both economic and environmental sustainability.

## 4. Role of Exogenous Nutritional Stimuli in Nutritional Programing in Fish

**Figure 2:**
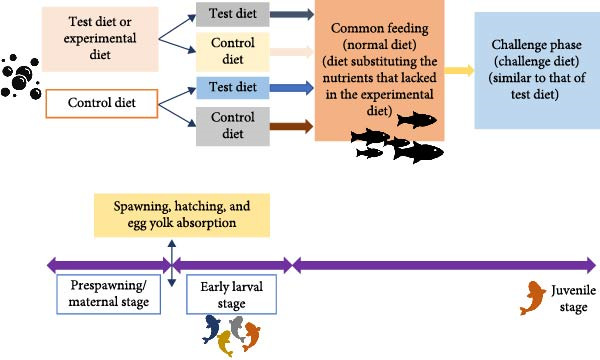
Procedure followed in nutritional programing of fishes.

Nutritional programing in fish is a phenomenon where early‐life nutritional exposures induce long‐term physiological adaptations, influencing metabolism, growth, and overall fitness. While maternal nutrition plays a significant role in shaping offspring development through yolk provisioning, exogenous nutritional stimuli—nutrients obtained from the environment posthatch—also contribute to programing metabolic pathways and digestive capacities (Table 2) in fish [5]. After yolk absorption, larval fish rely entirely on exogenous feed, and the quality, composition, and timing of first feeding can induce lasting metabolic and physiological changes. Early exposure to specific dietary components influences enzyme activity, digestive efficiency, and nutrient utilization. For example, studies show that early introduction of plant‐based diets in carnivorous fish species improves their ability to digest and metabolize plant‐derived nutrients later in life, reducing dependence on marine‐based protein sources [37]. Digestive enzyme expression is particularly responsive to exogenous nutritional stimuli. During early development, pancreatic and intestinal enzyme activity is highly plastic, allowing fish to adjust to available nutrient sources. A study on rainbow trout (*O. mykiss*) revealed that larvae initially fed a plant‐protein‐based diet exhibited higher amylase and protease activity as juveniles, demonstrating long‐term adaptation to carbohydrate‐rich feeds [22]. Similarly, European sea bass (*D. labrax*) larvae exposed to diets high in lipids during early feeding showed persistent increases in lipid metabolism and fatty acid oxidation rates [9]. Exogenous nutrition during early development influences metabolic programing by modulating the expression of genes involved in energy metabolism. High‐protein or high‐lipid diets in larval fish can alter the expression of genes regulating glucose homeostasis, lipid storage, and mitochondrial function. In Senegalese sole (*S. senegalensis*), early exposure to high‐lipid diets resulted in increased expression of genes related to fatty acid β‐oxidation, allowing for improved lipid utilization in juveniles [28]. Additionally, fish reared on early carbohydrate‐rich diets exhibited higher glycogen storage capacity, improving energy balance during fasting periods [32]. Epigenetic modifications further reinforce these metabolic adaptations. DNA methylation, histone modifications, and microRNA regulation play key roles in maintaining metabolic flexibility. For instance, early dietary interventions in zebrafish altered DNA methylation patterns in genes responsible for lipid metabolism, influencing fat deposition and energy efficiency later in life [35]. Nonetheless, fish may retain heightened sensitivity to nutritional programing, as evidenced by stimuli received from the maternally derived nutrients in the yolk or from the diet of the larvae, even after the depletion of the yolk supply [32]. Although developmental processes differ substantially between mammals and fish, comparative experimental frameworks, such as those illustrated in Figure 2 can provide valuable insights for understanding the underlying mechanisms of nutritional programing in fish and their broader relevance to biomedical and aquaculture research.

**Table 2:** Summary of the fish nutritional programing studies evidenced with context to exogenous nutritional stimuli.

Species	Critical window for programing	Effects	Nutritional stimulus	References
Atlantic cod	Larval feeding	Growth	Copepod	[52–54]
	Larval feeding	Stress tolerance	Copepod	[54]
Rainbow trout	Larval feeding	Growth, muscle growth	High fat	[55]
	Larval feeding	Carbohydrate metabolism	Hyperglucidic + hypoxia	[56, 57]
	Larval feeding	Carbohydrate metabolism	Hyperglucidic	[6, 12]
	Prior to spawning	Growth, survival; ingestion	Methyl group donor	[58]
	Prior to spawning	Lipid metabolism; carbohydrate metabolism	Methyl group donor	[59]
	Adult life cycle	Lipid metabolism; carbohydrate metabolism; muscle growth	Plant‐based diet	[60]
	Larval feeding	Ingestion	Plant‐based diet	[22]
	Larval feeding	Growth	Plant‐based diet	[22, 61]
	Larval feeding	Lipid metabolism; muscle metabolism	Vitamin supplementation	[62]
European seabass	Larval feeding	Lipid metabolism	HUFA deficiency	[63, 64]
	Larval feeding	Growth	Hyperglucidic	[65]
	Larval feeding	Stress tolerance	Hyperglucidic	
	Larval feeding	Hypoxia tolerance	Hyperglucidic	
	Larval feeding	Carbohydrate metabolism	Hyperglucidic	
Siberian sturgeon	Larval feeding	Carbohydrate metabolism	Hyperglucidic	[66]
Gilthead seabream	Larval feeding	Carbohydrate metabolism	Hyperglucidic	[66]
	Larval feeding	Growth, digestion	Plant‐based diet	[67]
	Larval feeding;	Inflammation	Plant‐based diet	[67, 68]
	Juvenile	Lipid metabolism, growth	Plant‐based diet	[37]
Zebrafish	Embryonic stage	Carbohydrate metabolism	Hyperglucidic	[69, 70]
	Larval feeding	Carbohydrate metabolism	Hyperglucidic	[7, 71]
	Adult life cycle	Lipid metabolism	Maternal one‐carbon micronutrient deficiency	[35, 36]
	Adult life cycle	Lipid metabolism	Maternal high ARA	[72, 73]
	Larval feeding	Inflammation	Plant‐based diet	[67, 68]
Senegalese sole	Larval feeding	Growth	Intact protein (vs hydrolysate with polypeptides)	[74]
Ballan wrasse	Larval feeding	Growth, muscle growth	Copepod compared to rotifer as a control	[54]
Pike perch	Larval feeding	Stress tolerance and Brain development	HUFA	[75]
Largemouth bass (*Micropterus salmoides*)	Larval feeding	Growth performance and gut microbiome	Formulated soybean meal‐based or soy saponin‐supplemented diet	[76]
Yellow croaker (*Larimichthys crocea*)	Larval feeding	growth performance, lipid metabolism, and antioxidant capacity	Vegetable oil‐based diet	[33]
Medaka fish (*Oryzias latipes*)	Larval feeding	Reversible and persistent epigenetic changes in medaka hepatocytes	High‐fat diet	[77]
Atlantic salmon (*Salmo salar*)	First exogenous feeding	Growth, lipid metabolism	Vegetable‐based diet versus standard marine‐based control	[78]
	First feeding	Gut microbiota composition and metabolic capacity	Plant‐based diet	[79]
Sterlet sturgeon (*Acipenser ruthenus*)	Larval feeding	Growth performance, body composition, and gene expression (GH, IGF‐1, Ghrelin)	Diets with varying levels of Soybean meal	[80]
Rainbow trout	Juvenile stage	Growth and liver lipid metabolism	Zinc nutrition	[81]

### 4.1. Prolonged Effects of Initial Feeding Regimens on Ichthyic Growth

The correlation between early life nutrition and growth was first documented in the 1960s [82], and there has been a continuous accumulation of supporting evidence. Studies have revealed that maternal protein and caloric restriction can lead to impaired growth in rats after birth, following standard feeding practices [83, 84]. Instances of early malnutrition during late gestation and lactation in rats have steadily resulted in reduced body weight, delayed puberty onset, and lower weight at puberty initiation [85]. These alterations in the growth curve have long‐lasting implications for overall health. Moreover, various studies indicate that the initial feeding regimen can influence the long‐term growth of fish species. For example, Atlantic cod (*Gadus morhua*) and Ballan wrasse (*Labrus bergylta*) larvae exhibited accelerated growth when fed zooplankton (primarily copepods) at their first feeding compared to rotifers (*Brachionus plicatilis*) or *Artemia* sp., and this positive impact continued into their juvenile stage even after an extended period of standard feeding [52–54]. In a study involving Senegalese sole (*S.senegalensis*), larvae fish fed intact protein at their first feeding, as opposed to protein hydrolysates made up of peptides, displayed higher dry weight after a month of common feeding [74]. Nonetheless, some studies have reported similarity in growth parameters, despite larger body sizes in the experimental group [52, 54, 74]. The question remains whether the growth‐promoting effects observed with zooplankton and intact protein diets or high carbohydrate‐based diets represent a programed response or a transient effect that may diminish with an extended period of common feeding [86].

### 4.2. Fish Neural Development via Nutritional Programing at Early Larval Stage Feeding

Early‐life dietary interventions can modulate gene expression patterns in the brain, with lasting consequences for neural growth, synaptogenesis, and cognitive functions. Nutritional programing—where early dietary interventions induce long‐lasting physiological effects—has emerged as a promising strategy to influence neural development and cognitive functions in fish. During the larval stage, the brain undergoes rapid growth and differentiation, making it highly sensitive to nutritional inputs (Table 3). Essential nutrients such as long‐chain PUFAs (LC‐PUFAs), particularly DHA, have been shown to support the formation of neural membranes and synaptic connectivity in fish species, including Atlantic salmon (*Salmo salar*) and zebrafish (*D. rerio*) [87, 90, 96]. Deficiencies in these key lipids during early development can result in impaired neural function, reduced learning ability, and lower survival rates [88]. Also, amino acids, especially taurine and tryptophan, play pivotal roles in brain development, acting as neurotransmitter precursors and influencing the serotonergic system [89]. Early dietary inclusion of such nutrients can modulate the expression of genes related to neural differentiation and neurotransmission [92]. Furthermore, feeding schedules and nutrient delivery modes (e.g., live feed enrichment vs microdiets) also influence the bioavailability of critical neuroactive nutrients, potentially affecting neural circuit formation and sensory development [8]. NP using DHA‐rich microdiets during the larval phase enhances the expression of neurotrophic factors such as brain‐derived neurotrophic factor (BDNF) and nerve growth factor (NGF), both of which are crucial for brain cell proliferation and differentiation [97]. Nonetheless, the neural development of fish concerning nutritional programing remains inadequately explored, although a single study suggests that nutrition during the larval phase can have enduring implications. Larval pikeperch (*Sander lucioperca*) were raised on either a diet lacking in DHA or a diet supplemented with DHA, followed by an extended period of common feeding. The juveniles originating from the DHA‐deficient larval diet exhibited a reduced cephalic index (brain mass/body mass × 100) compared to those fed the DHA‐supplemented diet, along with lower brain DHA content [75]. Consequently, the early larval stage continues to be recognized as a pivotal period for the targeted administration of neuroactive nutrients, aiming to optimize neural development and improve the organism’s adaptive potential as research in fish nutritional programing advances.

**Table 3:** Categorized studies on nutritional programing and neural development in fish larvae.

Focus area	Fish species	Nutritional intervention	Main outcomes	References
Essential fatty acids and brain development	Atlantic salmon (*Salmo salar*)	DHA/EPA‐enriched diets during early larval feeding	Improved neural membrane integrity and brain development	[87]
LC‐PUFA deficiency impact	Sea bass (*Dicentrarchus labrax*)	Low LC‐PUFA diets in larvae	Impaired learning ability and altered brain lipid profiles	[88]
Amino acid supplementation	Rainbow trout (*Oncorhynchus mykiss*)	Early tryptophan and taurine supplementation	Modulated serotonergic system and stress response	[89]
Epigenetic reprograming	Rainbow trout (*O. mykiss*)	High‐carb diet during larval stage	DNA methylation changes affecting neural genes	[22]
Gene expression and brain development	pejerrey (*Odontesthes bonariensis*)	Varying protein sources during early feeding	Altered expression of neurodevelopmental genes	[90]
Early feed restriction	Rainbow trout (*O. mykiss*)	Early‐life feed restriction	Long‐term alteration in lipid metabolism and possible neural outcomes	[91]
Feeding mode and brain development	Atlantic cod (*Gadus morhua*)	Live feed vs. formulated microdiets	Differential uptake of neuroactive nutrients	[8]
Digestive physiology and nutrient absorption	Multiple marine species	Timing of first feeding and nutrient type	Indirect effects on neurodevelopment via improved nutrient uptake	[92]
Parental nutritional programing	Yellow catfish (*Tachysurus fulvidraco*)	Parental high‐carbohydrate diet	Altered metabolome, increased glucose metabolism‐related gene expressions, and global DNA methylation in larvae	[29]
Maternal diet and offspring lipid metabolism	Red drum (*Sciaenops ocellatus*)	Distinct maternal diets	Changes in larval lipid and fatty acid compositions despite uniform post‐hatching diets	[64]
Early leucine programing	Zebrafish (*Danio rerio*)	Early leucine immersion	Enhanced mTOR signaling and protein utilization via DNA methylation	[93]
Soybean meal and gut microbiota	Zebrafish (*D. rerio*)	Early exposure to soybean meal	Improved plant protein utilization and altered gut microbiota composition	[94]
Amino acid supplementation and thermal challenge	Zebrafish (*D. rerio*)	In ovo arginine or glutamine supplementation	Modulated metabolic responses and enhanced adaptation to higher temperatures	[95]

### 4.3. Reprograming Lipid Pathways: Exogenous Nutritional Stimuli Shaping Progeny Metabolism in Teleost

Exogenous nutritional stimuli during key developmental stages in teleost fish, such as broodstock conditioning and the early larval phase, have been shown to reprogram lipid metabolic pathways with long‐lasting effects on offspring physiology. For instance, broodstock diets enriched with highly unsaturated fatty acids (HUFAs) not only result in the deposition of specific lipids into eggs but also influence maternal mRNA and lipoprotein content, setting the stage for the offspring’s lipid metabolism [87, 98]. These maternally transferred lipids, particularly phospholipids delivered through yolk syncytial layer lipoprotein receptors, establish a lipid‐rich environment that primes embryonic hepatocytes for effective lipid metabolism and supports normal organ development [99]. In Atlantic salmon, progeny of broodstock supplemented with DHA show enhanced hepatic expression of genes like fatty acyl desaturase (FADS2) and elongase (Elovl5), which are critical for endogenous HUFA synthesis and maintaining membrane fluidity for proper cell signaling [24]. Similarly, early larval feeding with HUFA‐enriched diets upregulates genes such as carnitine palmitoyltransferase I (CPT1) and PPAR alpha (PPARα), steering metabolism towards lipid oxidation and efficient energy use [100]. Notably, these metabolic changes are driven by DNA methylation and histone modifications at lipid metabolism‐related gene loci, which enable lasting gene expression changes beyond the initial exposure [62, 101]. For example, increased histone H3K27 acetylation at the fatty acid synthase (FAS) gene enhances chromatin accessibility and supports sustained lipogenic gene transcription. MicroRNAs such as miR‐122, miR‐33, and miR‐27 further regulate lipid metabolism by targeting related mRNAs, thereby refining lipid homeostasis [101]. These early programing effects result in significant phenotypic benefits. Zebrafish and seabream offspring demonstrate improved tissue deposition of n‐3 HUFAs, accelerated growth, and enhanced stress tolerance. Functional assessments also show increased feed conversion ratios and improved resilience to hypoxic conditions [12, 34]. Early nutrition also influences adipocyte function by regulating perilipin protein expression, supporting lipid droplet formation and energy storage [102]. Environmental factors, such as water temperature, interact with nutritional programing; fish raised in cooler water exhibit higher expression of CPT1 and PPARα if they were previously fed HUFA‐rich larval diets [103]. Further evidence of nutritional programing comes from studies in European seabass. Larvae initially fed low or high HUFA diets were later challenged with a HUFA‐deficient diet. Interestingly, those previously fed low HUFA diets retained higher DHA levels in polar lipids and showed increased expression of the ∆6 desaturase (∆6D) gene, indicating metabolic adaptation to compensate for dietary deficiencies [63, 104]. Similar findings have been reported in red drum (*S. ocellatus*), where different maternal diets led to eggs and larvae with distinct fatty acid profiles. Even when larvae were fed the same diet post‐hatching, those from shrimp‐enriched maternal diets showed differences in fatty acid biosynthesis pathways and triglyceride accumulation, suggesting maternal influence on early lipid metabolism and potential effects on survival and behavior [64]. In large yellow croaker, early‐life exposure to vegetable oil (VO) versus FO diets resulted in long‐term metabolic differences. Fish initially fed VO diets had lower liver lipid accumulation, triglyceride levels, and serum free fatty acids when later challenged with a VO diet again. These outcomes were supported by changes in lipogenesis‐related gene and protein expression, indicating a protective effect of VO nutritional programing against diet‐induced liver issues [33]. Overall, nutritional programing in teleosts via broodstock and early larval diets reshapes lipid metabolism through maternal provisioning, gene expression, epigenetic reprograming, and environmental interactions. These early interventions improve growth, metabolic efficiency, and health, offering promising strategies for optimizing performance and sustainability in aquaculture [62].

Although research on the impact of maternal diet on nutrient retention in young fish is limited, various studies indicate that the diet provided at first feeding influences nutrient retention. For instance, juvenile zebrafish exhibited changes in peptide absorption and transport of transport after being fed a soybean meal (SBM) diet for 3 days during their initial feeding, implying that early dietary choices may program intestinal responses [67]. In the intestinal mucosal cells, dietary lipids are primarily re‐esterified and incorporated into chylomicrons or very low‐density lipoproteins (VLDLs). Enhanced gut clearance, encompassing the absorption and movement of chylomicrons and VLDL, might facilitate the intake and assimilation of dietary fatty acids [105]. Conversely, a decrease in the efficiency of fatty acid absorption and transport, such as in chylomicron synthesis, may lead to the accumulation of lipid droplets within gut enterocytes, thereby hindering further absorption and assimilation [106]. Triglycerides in chylomicrons and VLDL are subsequently hydrolyzed by lipoprotein lipase (lpl) at peripheral tissues, facilitating their absorption for either catabolism or storage [107, 108]. Turkmen et al. [37]. discovered that a maternal diet rich in linseed oil (which is low in HUFAs) resulted in a downregulation of hepatic lpl expression in the offspring of adult gilthead seabream. This reduction in lipid deposition in the liver likely accounts for the observed decrease in liver lipid content [37]. Additionally, diminished lipoprotein synthesis in the liver could lead to reduced transport of lipids to extrahepatic tissues, resulting in the accumulation of lipid vacuoles in hepatocytes and an increase in lipid storage [109]. Thus, it is plausible that early nutritional interventions can program nutrient retention in offspring by influencing lipoprotein metabolism.

### 4.4. Carbohydrate Metabolism

Prenatal protein restriction led to a decrease in glucokinase (GK) and an increase in phosphoenolpyruvate carboxykinase‐1 (PEPCK‐1) activities in the livers of rodent progeny during weaning and adulthood [110]. The expression of GK and PEPCK occurs in distinct liver regions. Thus, a potential rationale for the contrasting activity changes could be the long‐term modification of the liver’s physical structure and function due to inadequate prenatal nutrition [110]. Notably, GK and PEPCK are not expressed until postpartum, yet their regulation is influenced by prenatal nutrition, implying that programing takes place before transcription. Hales et al. [111] similarly documented lower pancreatic GK activity in rodent progeny (3 months old) whose mothers were on a low‐protein diet throughout both pregnancy and lactation. These offspring displayed decreased glucose tolerance as adults, with elevated glucose levels in the blood serum after glucose administration into the intraperitoneal cavity.

**Figure 3:**
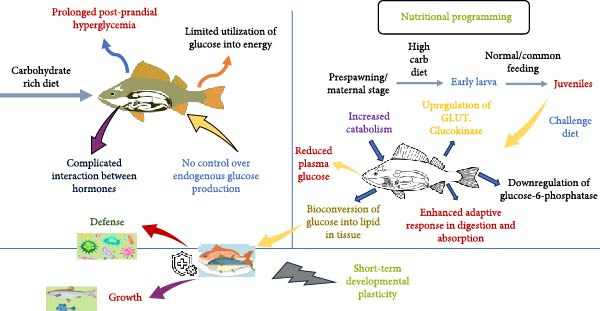
Concept of nutritional programing: Tailoring specific metabolic pathways or functions in fish, to improve the use of dietary carbohydrates.

The notion of programing through nutrition introduces the prospect of customizing explicit pathways related to metabolism or functions to enhance the utilization of dietary carbohydrates in fish (Figure 3). The fundamental idea, according to Geurden et al. [6], is that a dietary stimulus applied during crucial developmental phases in initial stages of life could have enduring impacts on physiological functions in growth and adult stages. Consequently, there are various studies where the feasibility of an intense short‐term hyperglucidic stimulus in early life to establish an adaptive capacity to manage a high carbohydrate diet challenge in later phases has been experimented [6, 7, 12, 66, 69, 70, 112]. The adaptability toward early programing of carbohydrate metabolism seemed to be more noticeable in zebrafish and sturgeon [66, 112] when compared to carnivorous rainbow trout [12]. Furthermore, the metabolic programing concept showed success in altering digestive enzymes (resulting in increased expression of amylase and maltase during a challenge test in juveniles) in rainbow trout [6]. Likewise, a study by Hou and Fuiman [7]. using tracers indicated that repetitive high‐glucose stimuli during early stages (larvae) could possibly impact oxidation of glucose and de novo lipogenesis in gilthead seabream. Overall, these aforementioned studies also emphasized that the source, the duration, and intensity of the dietary hyperglucidic stimulus, as well as the timing of administration that is, the critical transition window during which the stimulus is administered, are crucial factors that likely influence the success of nutritional imprinting. For example, a study demonstrated that a glucose stimulus during the embryonic stage (microinjection of 2M glucose into the yolk 1 day after fertilization, i.e., 1 dph) could lead to enduring changes in the metabolism of juvenile zebra fish. These changes are associated with an increased capacity for glucose oxidation in peripheral tissues and a decrease in hepatic glucose production when these fish are later exposed to a carbohydrate‐rich diet [70]. However, a similar glucose stimulus at 0.2 days postfertilization had minimal effects on the long‐term regulation of genes related to carbohydrate metabolism in zebrafish [69], underscoring the significance of administering nutritional stimuli at the most suitable developmental stage. Moreover, long‐term impacts of varying dietary carbohydrate levels on glucose metabolism in the omnivorous Nile Tilapia (*Oreochromis niloticus*) were performed. The fish were fed from the initial feeding stage until they reached adulthood over a period of 40 weeks, with three distinct carbohydrate levels (dextrin): 0% (CHO‐L), 30% (CHO‐M), and 50% (CHO‐H). Assessments were made on growth performance, blood metabolite parameters, the proximate composition of the entire body, as well as muscle and liver tissues, in addition to mRNA levels for genes associated with glycolysis, gluconeogenesis, lipogenesis, and glucose transport in both liver and muscle tissues at weeks 26 and 40. The results indicated that fish on the CHO‐M diet achieved optimal growth performance, whereas those on the CHO‐H diet exhibited the least growth. These findings imply that a specific level of dietary carbohydrates can be advantageous for Nile Tilapia. However, excessively high carbohydrate levels, particularly when accompanied by insufficient dietary protein, do not support growth. Fish consuming carbohydrates showed a notable increase in lipid accumulation across the whole body, muscle, and liver tissues, likely due to enhanced lipogenic capacity, as evidenced by elevated hepatic FAS mRNA levels and activity, along with increased plasma triglyceride levels. Nile Tilapia appear to adapt effectively to carbohydrate intake, as indicated by (i) mild postprandial hyperglycemia (5.3–6.1 mM), (ii) elevated hepatic glycogen levels, (iii) increased glycolytic pyruvate kinase enzyme activity in muscle (both mRNA levels and activity), and (iv) suppression of the gluconeogenic pathway for glucose‐6‐phosphatase and phosphoenolpyruvate carboxykinase enzymes at both molecular and enzymatic levels. Collectively, these findings suggest that the recognized differences in the ability of carnivorous and omnivorous fish species to utilize dietary carbohydrates may be associated with variations in the regulation of gluconeogenic and lipogenic pathways [113]. Thus, it is also imperative to systematically investigate the impact of single stimuli compared to repeated nutritional stimuli and fine‐tune the duration, intensity, and quantity of the stimulus for each target species. In addition to the stimulus and the timing of application, future studies should facilitate a deeper comprehension of the biological mechanisms that imprint the nutritional event until adulthood, such as adaptive alterations in cellular phenotype (epigenetic phenomenon), nutrient‐responsive signaling pathways, or even gut microbiota. Alternatively, the potential carryover effect of utilizing broodstock diets containing insulin‐sensitizing quasivitamins (myoinositol) and trace elements (chromium) to positively influence glucose metabolism in offspring warrants further exploration.

## 5. Mechanisms of Nutritional Programing on Ingestion in Fish

Feeding is a multifaceted activity encompassing not only the act of food consumption itself but also behaviors related to searching for food and the drive to eat. The regulation of feeding ultimately lies within the central feeding centers of the brain, which interpret signals from hormones originating from both the brain and the periphery. These signals include hormones that either stimulate feeding behavior, such as orexin and neuropeptide Y (NPY), or suppress it, such as cocaine‐ and amphetamine‐regulated transcript (CART) and proopiomelanocortin (POMC) [114–117]. The functioning of these feeding centers is also influenced by metabolic and neural signals from the periphery, which provide feedback on meal consumption and nutritional status. Significant changes in the structure and function of the hypothalamus have been extensively documented in response to early nutritional cues in biomedical literature. Studies in mammals have demonstrated that nutritional manipulations during early development can lead to long‐term metabolic and appetite‐regulating changes. For instance, rats raised in small litters (resulting in postnatal over‐nourishment) exhibit increased body weight, hyperinsulinemia, obesity, and other metabolic irregularities. Additionally, changes in the activity of hypothalamic neurons in response to neuropeptides indicate a heightened orexigenic drive and decreased anorexigenic signaling [118]. While limited research has explored the feeding behavior of fish in a programing context, dietary methionine levels in rainbow trout (*O. mykiss*) broodstock have been shown to impact the survival and growth of their offspring. This effect may be linked to changes in the expression of POMCa (an anorexigenic peptide) and NPY (an orexigenic peptide) in the offspring post‐hatching, with the influence on POMCa expression persisting for 3 weeks after the commencement of exogenous feeding [58]. However, some studies did not find significant effects on the feed intake of juvenile rainbow trout or Siberian sturgeon following early exposure to high carbohydrate stimuli [6, 66].

### 5.1. Neuroendocrine Regulation of Feeding Behavior

Feeding behavior in fish is primarily regulated by the hypothalamic–pituitary axis, which integrates signals from peripheral tissues to regulate appetite. Early dietary exposure influences the expression of appetite‐regulating hormones, leading to long‐term modifications in feeding behavior. Fish larvae exposed to high‐carbohydrate diets early in development show a persistent upregulation of NPY and AgRP, which promote food intake, while those exposed to high‐protein diets exhibit increased expression of CART, which suppresses appetite [32]. These hormonal adjustments persist into juvenile and adult stages, programing feeding behavior based on early nutrient availability.

### 5.2. Sensory Adaptations and Food Preference

Nutritional programing also modifies sensory perception, affecting food preference and palatability responses. The gustatory and olfactory systems in fish are highly plastic during early development, and dietary exposure during this period can influence long‐term feeding choices. Research in mammals has shown that perinatal exposure to certain chemical cues can alter natural flavor preferences. Rodent progeny exposed to inherently aversive odors or flavors through maternal diet or early post‐weaning stages displayed enhanced acceptance and voluntary consumption of these substances [119, 120]. A similar phenomenon has been observed in fish, where increased consumption of plant‐based diets by rainbow trout was attributed to either a shift in flavor preference or a reduced aversion to the food’s flavor due to early exposure to plant materials [22]. A subsequent study utilizing microarray analysis revealed that the plant‐based diet influenced pathways associated with flavor recognition and feeding preferences [121].

### 5.3. Metabolic Signaling and Energy Homeostasis

Dietary programing affects metabolic signaling pathways that regulate energy balance and feeding motivation. IGFs and leptin play critical roles in mediating these effects. Fish fed high‐lipid diets during early stages show increased leptin sensitivity, leading to reduced feeding rates and enhanced lipid oxidation efficiency later in life [28]. Conversely, carbohydrate‐enriched diets in larvae result in a metabolic preference for glucose as an energy source, altering the activity of glucose transporters and glycolytic enzymes [32]. These metabolic adjustments influence ingestion patterns by aligning feeding behavior with the programed metabolic capacity for nutrient utilization.

### 5.4. Appetite Regulation in Larval Fish

The mechanisms governing appetite regulation in larval fish are still not fully understood. It remains unclear when the internal regulatory mechanisms of appetite become fully functional. Larval fish exhibit continuous feeding behavior in the presence of prey, suggesting that satiety signals produced by the gastrointestinal tract (GIT), known as anorexigenic factors, may not yet be fully developed [92]. The involvement of appetite‐regulating factors varies significantly among species [115], making it necessary to exercise caution when extrapolating findings from mammals or other fish species to predict the impact of dietary programing on feeding behavior.

### 5.5. Implications for Aquaculture

Understanding the mechanisms behind dietary nutritional programing on ingestion has significant applications in aquaculture. By optimizing early feeding strategies, fish can be conditioned to accept and efficiently utilize alternative protein sources, such as plant‐ or insect‐based feeds, reducing reliance on fishmeal and enhancing aquaculture sustainability [5]. Additionally, dietary programing can improve feed efficiency, reduce waste production, and enhance overall fish growth and health. In conclusion, dietary nutritional programing influences ingestion in fish through neuroendocrine regulation, sensory adaptation, and metabolic signaling. Early dietary interventions shape long‐term feeding behavior, improving dietary flexibility and metabolic efficiency. Further research is needed to refine feeding protocols and maximize the benefits of nutritional programing in aquaculture.

## 6. Mechanisms of Nutritional Programing on Digestion and Absorption in Fish

The recent surge of interest in fish nutrition as a scientific field has prompted a shift in research focus. Historically, studies up to the mid‐twentieth century primarily delved into the anatomy of fish digestive systems and their physiological behaviors in natural environments. However, the increase in fish farming has highlighted the vital need to investigate the dietary needs of fish. This is essential as diets play a pivotal role in influencing various aspects such as behavior, structural integrity, overall health, physiological functions, reproductive patterns, and growth trajectories [122].

### 6.1. Digestion and Nutrient Absorption in Fish

The nutrients consumed by fish undergo intricate processes of digestion and absorption before being metabolized. Digestion is a complex interplay of physical, chemical, and enzymatic mechanisms that commence upon food intake and culminate in excretion. The enzymatic secretions play an active role in the breakdown of dietary components via hydrolysis. Enzymatic processes are highly specific, targeting the hydrolysis of proteins, carbohydrates, lipids, and nucleic acids with the aid of numerous enzymes [123]. However, the effects of programing on the GIT or digestive function are still largely unknown at this point, except for the endocrine pancreas. Studies have shown that reduced activities of digestive enzymes such as maltase, aminopeptidase M, and glycyl‐leucine dipeptidase in the intestines of adult rat offspring were linked to maternal protein deficiency during pregnancy and lactation [124]. Furthermore, the GITs of newborn animals have ontogenetic adaptations to their environment, including the ability to digest and adapt to various foods [125]. Nevertheless, Guilloteau et al. [126] have cast doubts on the suitability of rodents as models for investigating GIT programing due to disparities in their GIT development and digestive functionality compared to humans.

### 6.2. Enzymatic Development and Nutritional Influence

Most fish species exhibit detectable levels of digestive enzymes early in the weaning phase [127] or upon hatching [128, 129], in spite of the incomplete maturation of their digestive system. The activation of specific digestive enzymes occurs at various developmental stages [130–132], a process that is influenced by nutrient availability [129]. Juvenile rainbow trout, after being exposed to a high‐carbohydrate diet during the larval stage, demonstrated increased expression of pancreatic α‐amylase and intestinal maltase when faced with a similar diet challenge [6]. Consistent with these findings, research on common carp has shown that nutritional history significantly influences feed efficiency and protein utilization in later life stages, highlighting the beneficial physio‐biochemical changes brought about by initial feeding [30]. Adult zebrafish, which were initially subjected to high glucose levels during early feeding, displayed heightened expression and functionality of α‐amylase when tested under challenging conditions [112]. Gilthead seabream larvae that received SBM as their diet for 2 weeks post‐first feeding exhibited reduced activities of pancreatic enzymes (trypsin, chymotrypsin, and amylase) and experienced inhibited growth. Following the removal of SBM from their diet, these larvae resumed chymotrypsin and amylase activities; however, trypsin activity and growth did not recover [67].

### 6.3. Absorption and Transport Mechanisms

In the case of absorption and transport, after 3 weeks following common feeding, young zebrafish that consumed the SBM diet exhibited increased transportation of fatty acids and reduced absorption of peptides. This was characterized by the elevation of fabp2 expression and the suppression of slc15a1 levels in the intestines. Perera and Yu´fera [67] proposed that the GIT is likely to be influenced by the early dietary regimen.

### 6.4. Mechanisms of Nutritional Programing

Nutritional programing in fish digestion is a critical area of study that explores how early nutritional experiences can induce long‐term physiological adaptations, influencing growth, metabolism, and overall health. This concept, derived from developmental biology, suggests that early‐life nutritional interventions can shape digestive efficiency and metabolic pathways, impacting fish performance in aquaculture [22]. Feeding larvae with specific nutrient‐enriched diets can lead to long‐term changes in digestive enzyme activity and nutrient transporter expression, optimizing feed efficiency [133]. The gut microbiota also plays a crucial role in nutritional programing. Early exposure to different dietary components can shape the microbial community structure, influencing digestion and immunity. Studies have shown that early probiotic supplementation can establish a beneficial microbial balance, enhancing gut health and digestive efficiency in fish [134]. The microbiota interacts with the host through metabolic signaling, affecting nutrient absorption and energy metabolism. Certain microbial metabolites can act as epigenetic regulators, reinforcing the programing effects induced by diet [135].

Digestive enzyme activity is another key aspect of nutritional programing. Early nutritional interventions can induce long‐lasting modifications in the expression and activity of digestive enzymes such as proteases, amylases, and lipases. For instance, early exposure to high‐protein diets can enhance protease activity, improving protein digestion efficiency in later life stages [12]. Similarly, feeding fish with carbohydrate‐rich diets during early development can upregulate amylase expression, facilitating carbohydrate utilization [136].

## 7. Mechanisms of Nutritional Programing on Oxidation and Biosynthesis in Fish

The oxidation of fatty acids plays a crucial role as a significant energy source and a fundamental process in maintaining energy balance within the body. The supply of energy is facilitated through the β‐oxidation of unbound fatty acids that are transported to the mitochondria in the form of fatty acylcarnitine esters by carnitine acyltransferases, such as carnitine palmitoyl transferases [137]. Research indicates that alterations in mitochondrial oxidation are linked to the development of metabolic syndrome. Substituting FO with VO modifies the fatty acid composition in the liver and muscles, impacting the capacity for β‐oxidation and controlling the expression of cptI and cptII genes [138–140]. In marine species, the capacity for β‐oxidation can be influenced by the maternal diet or the initial feeding regime. For instance, feeding gilthead seabream broodstock a linseed oil‐based diet led to a decrease in cptIb expression in the offspring’s liver [37]. Similar effects were observed in juvenile rainbow trout when provided with vitamin supplementation during their initial feeding [62]. It has been proposed that changes in dietary fatty acid availability, instead of direct stimulation of the β‐oxidation pathway, primarily drive the altered oxidation resulting from dietary interventions [141]. The positive correlation between cptIb expression and the fatty acid 18:1(n‐9) in gilthead seabream supports the notion that monounsaturated fatty acids like 18:1(n‐9) are commonly utilized as energy sources. However, there is a growing body of contradictory evidence. Dysfunctional mitochondrial activity has been proposed as the fundamental molecular cause for the decreased fatty acid oxidation in the liver observed in the offspring of obese parents consuming a high‐calorie diet, as evidenced by multiple markers of hepatic mitochondrial function [142]. Additionally, another study found that the offspring of rats subjected to a deficiency of methyl donors during gestation and lactation displayed compromised mitochondrial fatty acid oxidation, characterized by the downregulation of essential enzymes related to mitochondrial fatty acid oxidation (HADHA, short‐chain acyl‐CoA dehydrogenase) and diminished activity of complexes I and II [143].

Marine fish generally exhibit a reduced capacity to utilize terrestrial oils owing to their elevated n‐3 LC‐PUFA requirements and the limited activity of enzymes responsible for synthesizing n‐3 LC‐PUFA from the fatty acid precursors present in plant oils [144]. The substitution of FO with VO appears to pose greater challenges in marine fish compared to species more adept at efficiently utilizing dietary lipids. The higher LC‐PUFA biosynthesis ability observed in freshwater fish, as opposed to marine fish, may be attributed to variations in feeding behavior and nutrient intake, with marine fish benefiting from consistent access to sources rich in LC‐PUFAs throughout their lifespan [5, 103]. Variations among fish species have also been linked to the distinct evolution of specific genes involved in lipid biosynthesis. The conversion of Linoleic acid (LA, 18:2n‐6) and ALA (18:3n‐3) into n‐3 LC‐PUFAs involves several elongation and desaturation steps, resulting in the production of longer carbon chain fatty acids like EPA (20:5n‐3) and DHA (22:6n‐3). The initial stage of n‐3 LC‐PUFA synthesis in vertebrates is mediated by the fatty acyl ∆‐6‐desaturase enzyme (∆6) encoded by the fatty acid desaturase 2 gene (fads2), which introduces a double bond at a specific position in long‐chain fatty acids. Subsequently, LA (18:2n‐6) and ALA (18:3n‐3) can be transformed into 18:3n‐6 and 18:4n‐3, respectively, followed by conversion to 20:3n‐6 and 20 : 4n‐3 after an elongation step. Another desaturation step catalyzed by a fatty acyl ∆‐5‐desaturase enzyme (∆5) leads to the formation of 20:5n‐3 (EPA) and 20:4n‐6 (ARA) from these precursors. The evolution of fish desaturases varies across species, resulting in a diverse array of desaturases. Notably, the fads2 gene in zebrafish (*Danio rerio*) exhibits both ∆5 and ∆6 activities [67].

In the context of programing research, the biomedical literature supplies evidence for the connections between metabolic irregularities and the synthesis of PUFAs. Offspring of growth‐retarded rats exhibited diminished activity of ∆5 desaturase (∆5D) in hepatic microsomes due to maternal protein limitation [144]. Numerous investigations have indicated an alteration in fatty acid synthesis activities in fish resulting from programing, albeit mostly concentrated on the gene expression level. Enhanced levels of substitution of FO with linseed oil (higher ALA and LA levels) in broodstock diets led to progressively increased expression of the ∆6D gene in gilthead seabream larvae, except for notable repression of ∆6D expression in progeny from adults fed a solely VO diet [5, 103]. The increased biosynthetic activity endured until the juvenile stage (4 months old), as demonstrated by elevated ARA, EPA, and DHA levels and a decline in their precursors in the liver with a raised maternal dietary linseed oil level [103]. Upon reaching adulthood, larvae from the partially replaced groups exhibited downregulation of hepatic elongase (Elovl6) [37]. In Senegalese sole embryos, elevated transcript levels of genes associated with fatty acid biosynthesis, specifically elongase 5 (Elovl5), and in newly hatched larvae (Elovl5 and ∆4 desaturase (∆4fad)), were observed when broodstock consumed a standard diet, as opposed to a HUFA‐supplemented diet. This indicates an ability to regulate lipid metabolism that is operational from the embryonic stage [28]. However, the disparities were subsequently reversed, with heightened expression of ∆4fad at 2 dph (days post‐hatching) and both ∆4fad and Elovl5 at 7 dph in larvae from the HUFA‐supplemented broodstock diet. Young European seabass subjected to a HUFA‐deficient diet during the larval period displayed increased expression of ∆6D, and greater DHA content in polar lipids, when faced with a HUFA‐deficient diet, alongside upregulation of three forms of PPAR (PPARs, a, b, and c) [63, 104]. Furthermore, investigations in long‐snout seahorses facilitated a comparison of the potential nutritional programing impact of paternal or maternal nourishment, revealing seahorses as a valuable model [145]. In these analyses, mysids with high or low levels of LC‐PUFA were fed to males or females from 16 seahorse breeding pairs separately before mating for 5 months. As anticipated, progeny from females fed high LC‐PUFA were larger than those from females fed low n‐3 LC‐PUFA [145]. Notably, these studies also illustrated that the nutritional condition of the male determines the morphology and feeding behaviors of the offspring, regardless of the mother’s diet. Thus, poor nutritional quality in male diets pre‐conception and during pregnancy resulted in abnormally large and heavy offspring with reduced survival chances, even if females were provided a high‐quality diet [145].

## 8. Mechanism of Nutritional Programing on Epigenetic Modification

Epigenetic modifications regulate gene expression without altering the DNA sequence, allowing environmental factors such as diet to influence phenotypic traits across generations [146]. Let’s say, a study by Moran et al. [48]. demonstrated that brown trout (*S. trutta*) fed a salt‐enriched diet exhibited changes in global DNA methylation patterns, which were associated with improved seawater acclimation. These findings suggest that dietary interventions during early development can induce long‐lasting epigenetic changes that enhance environmental adaptability.

**Figure 4:**
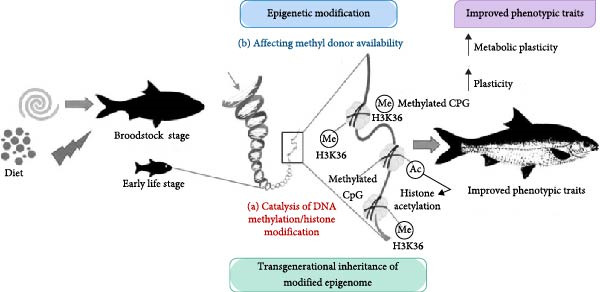
A schematic illustration depicting the potential uses of epigenetics in the nutritional programing of fish is presented. Nutritional stimuli can trigger alterations in gene expression via epigenetic mechanisms, such as DNA methylation and histone modifications, leading to phenotypic changes within the organism. These mechanisms may be associated with specific nutrient components or metabolites that function as regulators or substrates for enzymes responsible for catalyzing DNA methylation and histone modifications, or that influence the availability of methyl donors. The heritability of epigenetic markers and their corresponding phenotypes in subsequent generations underscores the significance of applying epigenetics in aquaculture, promoting more sustainable practices from both environmental and economic perspectives.

Epigenetic alterations that affect gene expression can be prompted by external cues, with the capacity to persist for a lifetime or across generations, ultimately impacting an individual’s phenotype. Recently, there has been a resurgence of interest in the potential impact of epigenetics on complex or quantitative characteristics. This surge in interest is partially fueled by the growing accessibility of high‐throughput sequencing techniques for investigating the epigenome. DNA methylation, the process by which a methyl group is attached to the C5 carbon of cytosine by DNA methyltransferase, has been the most extensively researched epigenetic mechanism so far, with delineation of the methodologies employed to acquire and analyze genome‐wide DNA methylation data. The impact of epigenetic mechanisms on the prediction of breeding values and the precision of genomic selection for enhancing the genetics of aquatic species is scrutinized. The plausibility of precisely regulating nutritional stimuli known to influence epigenetic processes to customize the growth of fish for aquaculture is also deliberated upon. Thus, enhanced comprehension of epigenetics and its effects on phenotype could enable more precise manipulation of fish development by carefully regulating various environmental factors (including fish diet) at different developmental stages. Nutrients and bioactive food components can alter epigenetic processes by directly inhibiting enzymes responsible for DNA methylation or histone modifications, or by modifying the availability of necessary substrates for these enzymatic reactions [147], a phenomenon referred to as nutritional programing (Figure 4). Although there is limited evidence of nutritional programing in fish [12, 112], if this phenomenon does occur in fish, it may be plausible to manipulate programing to enhance performance in aquaculture systems. The manipulation of the development of groups of larvae or fingerlings for programing could be easily and precisely accomplished as development occurs in the aquatic environment. Nutrients in the diet or other environmental factors may directly support development or induce changes in the epigenome that lead to varied gene expression levels post‐exposure, with some epigenetic alterations possibly being heritable across subsequent generations through paternal and/or maternal lineages. Furthermore, nutritional programing can impact growth by directly influencing the expression of genes involved in protein synthesis. Zhu et al. [93] showcased that early leucine programing could impact the methylation and expression of genes associated with protein synthesis in zebrafish.

DNA methylation and various other epigenetic regulations of gene expression are recognized to play a crucial role in modulating cellular differentiation in the course of development [148]. Numerous nutritional cues have been identified to influence the differentiation of fish cells during development, potentially by inducing alterations in the epigenetic imprints within specific cells. Deficiency in vitamins (A, D, and C) within sea bass has been demonstrated to interrupt the temporal sequence and coordination of growth factor gene expression, thereby impacting the differentiation of osteoblasts and leading to the conversion of some into adipocytes, resulting in deformities [149]. The conversion of osteoblasts to adipocytes can occur when the levels of dietary HUFAs are excessively high during the initial stages of marine fish development [65]. Salmon pre‐adipocytes exhibit the potential to differentiate into cells of the immune lineage (most likely macrophage‐like cells) contingent on environmental circumstances [150]. Nevertheless, the epigenetic signals that impact cellular differentiation during fish development have not yet been thoroughly explored.

The growth of skeletal muscle in fish is a dynamic process involving a blend of muscle fiber enlargement (hypertrophy) and the recruitment of new fibers (hyperplasia). The equilibrium between these processes dictates fiber density, subsequently influencing muscle texture and resistance to handling and processing [151, 152]. The hypertrophy‐hyperplasia equilibrium is influenced by genetic disparities among strains and external factors like early nutritional background [153]. The cellular advancement of fish muscle appears markedly influenced by the environmental conditions encountered by the egg, larvae, and fry throughout development. For instance, the temperature during embryonic phases has been observed to impact muscle fiber recruitment in zebra fish (*D. rerio*) and Atlantic cod (*G. morhua*) [154], and is also known to affect the emergence of deformities, abnormal heart development, and the expression of stress‐responsive genes in Atlantic salmon [155–157]. Despite the acknowledged importance of fry nutrition, research on the implications of fry nutrition for meat quality or health at harvest is relatively limited [158]. Conversely, most investigations on optimizing fillet quality have concentrated on the influence of dietary composition and feeding regimes for larger fish. The expression of several genes in the mature salmon fillet has been linked to fillet tenderness [159], yet there exists limited comprehension regarding the expression of these genes in early developmental stages, the factors influencing their expression, and the specific role, if any, that these genes might play in determining fillet quality [160].

Emerging discoveries indicate that fatty acids, specifically PUFA (n‐3 HUFAs), may have an impact on the epigenome [161]. The n‐3 HUFAs play a crucial role as precursors of eicosanoids, essential for the growth and development of fish [88], providing metabolic energy in the form of ATP [88], and contributing to membrane structure formation [144]. Alterations in the dietary lipid source of female salmon during gonadal development can influence the composition of egg fatty acids, potentially leading to abnormal embryonic development [162]. The supplementation of broodstock diets with higher levels of FO has been demonstrated to impact the survival rate of 3‐day posthatching larvae, egg characteristics, fry length, hatchling oil globule diameter, egg protein, and lipid content, as well as the morphology and metabolism of adipocytes in sea bream [163]. Furthermore, early life exposures can shape responses to nutritional cues in later life through epigenetic mechanisms [164], and exposure to high‐fat diets in early life stages could potentially predispose fish to obesity and related conditions, reduce resilience to pathogen exposure, and increase sensitivity to other stressors.

Consequently, the nutritional condition and health of the parents, eggs, larvae, or fry, along with the programing prior to conception or during early developmental stages, may influence the type, quantity, and distribution of osteoblast, adipocyte, and myocyte precursor cells, gene expression, and the ensuing development of muscle and meat quality [153], although additional research is necessary.

## 9. Nutritional Programing via a Crossover Developmental Strategy for Diet Adaptation

The aquaculture industry is predominantly reliant on freshwater fish farming, with a significant portion (86%) of production coming from freshwater rearing systems [165]. Traditionally, fish meal (FM) has been the preferred protein source due to its nutritional profile, but concerns about its sustainability and high cost necessitate the development of alternatives [166]. Plant‐based proteins offer a viable substitute; however, their high inclusion levels have been associated with reduced growth and feed efficiency, primarily due to antinutritional factors [167–170]. While the removal of these factors is possible, it is often economically unfeasible. Consequently, nutritional strategies that promote physiological adaptation to plant‐based diets, rather than altering feed composition, are gaining attention. Nutritional programing, the concept that early dietary experiences can induce lasting metabolic adaptations, has shown promise in aquaculture [76]. Fish development prior to hatching is largely influenced by maternal nutrition, with broodstock diet playing a critical role in embryogenesis and early larval development [40, 127]. However, the long‐term effects of broodstock nutritional history on progeny performance remain underexplored [24]. Thus, the study aimed to assess whether the complete substitution of FM with plant‐based diets affects juvenile performance in common carp and whether the broodstock’s nutritional history influences the progeny’s capacity to utilize such diets effectively.

In the study, complete replacement of FM and FO with plant‐based ingredients in broodstock diets negatively impacted the growth performance of larvae at 28 days after hatching (dah), suggesting that parental diet plays a critical role in shaping offspring development [5, 24]. Similar to findings in mammals [171], dietary HUFA deficiency in broodstock diets affected larval viability and growth, likely via changes in gene expression related to fatty acid metabolism, such as the Δ6‐desaturase gene.

During the fry stage, which serves as a transitional period between endogenous and exogenous feeding, the nutritional history of both broodstock and fry influenced final weight outcomes, even when specific growth parameters like SGR and CF were similar. This aligns with the concept that first‐feeding diets condition the fish’s metabolism, leading to persistent effects on nutrient utilization when the same diet is reintroduced later [76]. In crossover feeding experiments, fry performed better when re‐fed with their original diets than with complementary ones, supporting the idea that early diet exposure enhances later diet acceptance and utilization [121].

Notably, fish with an early history of fish‐based diets (F‐fish) showed greater flexibility in adapting to dietary changes compared to those with a plant‐based history (P‐fish). This flexibility was evident in both growth performance and metabolic enzyme activities (ALT and AST), which recovered in F‐fish but not in P‐fish upon return to their original diets. The diet‐specific efficiency in protein and energy utilization during different feeding phases highlights the critical role of early diet in shaping long‐term metabolic programing.

At the molecular level, the GH/IGF‐I axis, a central regulator of growth and nutrient partitioning in fish [172], was significantly influenced by nutritional history. Fish fed with diets mismatched to their early feeding experience showed downregulated hepatic expression of IGF‐I and GHR genes, correlating with reduced growth [173, 174]. These findings emphasize that nutritional programing can influence endocrine pathways, which in turn modulate growth and metabolic capacity during later life stages.

Overall, this study reinforces the premise that nutritional inputs during early developmental windows can induce long‐term metabolic and physiological adaptations in fish. Strategic manipulation of early diet—particularly via broodstock and larval nutrition—can therefore serve as a sustainable tool to enhance plant‐based diet utilization in aquaculture species [175].

## 10. Potential Limiting Steps of Programing use in Fish

The identification of specific nutritional constituents that influence programing in fish presents a multitude of complexities. First and foremost, the prevailing literature has engaged a wide range of experimental frameworks—such as adjustments in dietary interventions, initiation, and duration of nutritional stimuli—which complicates the discernment of common features. For instance, while it is generally acknowledged that VOs are characterized by low concentrations of long‐chain HUFAs and (n‐3) fatty acids, coupled with higher levels of short‐chain and (n‐6) fatty acids, the precise composition and essential fatty acid ratios differ significantly among various VO sources. This variability is further exacerbated by the disparate dietary incorporation levels implemented in different studies. Secondly, although the majority of investigations have concentrated on the programing effects associated with a singular class of nutrients, it is conceivable that concurrent variations in the dietary availability of nontarget macronutrients or micronutrients may arise due to dietary alterations, thereby necessitating consideration of the overall nutrient balance [176, 177]. A particular study has indicated that elevated blood pressure in individuals with low birth weights correlates with a diminished protein‐to‐carbohydrate ratio in the average diet during gestation, rather than any other absolute intake metrics [178]. Thirdly, controlling the precise nutrient intake by broodstock or larvae presents significant challenges. In terms of implementing dietary interventions for larvae, the nutritional profiles of live prey exhibit considerable variation contingent upon the culture methodologies employed, the enrichment products utilized, and the metabolic processes of the live prey organisms. For instance, a liposome‐based technique (which encapsulates water‐soluble nutrients within microparticles such as liposomes) has demonstrated efficacy in enriching live prey, yet the nutrient concentrations within these organisms may fluctuate based on the composition of the liposome membranes [179, 180]. Additionally, the metabolic activity of live prey (e.g., rotifers, copepods, and Artemia) may further modify their intended nutritional profiles, introducing additional variability into the nutritional intake of larvae [notably, a reduction in temperature has been proposed to mitigate the metabolic rate of rotifers and prolong nutrient storage [180]. For example, it has been observed that Artemia may retroconvert DHA into EPA, whereas rotifers tend to metabolize phospholipids and sequester the resultant digestive products as triacylglycerols (TAGs) [8]. These multifaceted factors contribute to the increasing complexity surrounding the identification of nutritional components that underlie the phenomenon of nutritional programing.

Early nutritional intake may interact synergistically with various environmental factors, including oxygen availability, temperature fluctuations, and pH levels, thereby eliciting differential metabolic responses in various organisms [100]. For instance, empirical evidence has demonstrated that a high‐carbohydrate dietary intervention during the embryonic and early larval phases can instigate modifications in carbohydrate metabolism in juvenile *O. mykiss*; however, when this dietary approach is coupled with hypoxic conditions, the resultant effects diverge, revealing a significant interaction between prior dietary exposure and oxygen concentration [56, 57]. Furthermore, research has indicated that early‐life nutritional programing in rainbow trout does not yield a discernible advantage in carbohydrate metabolism; indeed, it fails to produce homogeneous and consistent physiological responses, despite being indicative of similar physiological functions, as the modulation of physiological markers is not uniformly observed, nor are the same tissues or organs uniformly affected by these persistent alterations [60]. One might contemplate whether these disparate responses predominantly signify, at the onset of exogenous feeding in rainbow trout alevins, the presence of a buffering capability within the gene regulatory networks implicated in carbohydrate digestion and metabolism. Consequently, it raises the question of whether the timing of nutritional programing may not align appropriately with the developmental stage of this piscine species. Furthermore, it continues to be an unresolved question whether these diverse responses may lead to physiological trade‐offs, which could influence organismal‐level outcomes. These intricate responses have largely remained unexamined but are increasingly pertinent in light of the escalating frequency and diversity of environmental distress.

In addition, the comprehension of the epigenome and the alterations influencing specific phenotypes necessitates intricate experimental procedures utilizing familial material, akin to those experiments required for the identification of quantitative trait loci. The financial investment associated with the measurement of epigenetic modifications across the genome is progressively diminishing; however, supporting experimental expenditures and considerations must be addressed when investigating the epigenome. For example, at which developmental stage(s) should the epigenome be evaluated? To ascertain whether the effects are transmittable, it would be imperative to analyze the epigenome of mature progenitors and their offspring during an early developmental stage (egg or larvae). In certain instances, it may be essential to monitor epigenetic markers throughout the developmental trajectory of the fish, which would also necessitate repetitive assessments of the specimens. Presently, there exists a paucity of knowledge regarding the genes that are modulated by nutritional interventions during early life phases (e.g., fry) and the epigenetic mechanisms in these initial stages that influence robustness and meat quality. An understanding of these mechanisms could catalyze modifications in the formulation of novel diets, enhance selection precision for the genetic enhancement of significant traits, and lead to improved robustness alongside greater uniformity and quality at the time of harvest. If the programing endures until maturation, it may yield heritable modifications and potentially influence the early developmental stages of succeeding generations. Histone methylation may function as an epigenetic modulator in certain molluskan species [181]. Consequently, it is crucial to ascertain whether the programing endures until harvest and to elucidate the regulatory mechanisms governing programing in both fish and shellfish.

## 11. Future Prospect and Conclusion

Sustainable aquaculture necessitates a reduction in the utilization of fishery‐derived products in feed formulations, alongside at least a fractional substitution with more environmentally sustainable ingredients [5]. Nutritional programing represents a hopeful approach to enhance crop yields while simultaneously minimizing economic expenditures and ecological repercussions [100, 182]. This enhancement may perhaps be realized via modifying the dietary regimen of broodstock or implementing nutritional interventions during the larval developmental stage. Current research within aquaculture practices is driven by the physical and functional adaptability exhibited by fish in response to nutritional programing and has effectively employed this methodology across several economically significant fish species. Additional investigations are warranted to ascertain the longevity of the programing effects established through this approach and to evaluate any potential adverse consequences. The exploration of nutritional programing in fish is in its nascent stages, and numerous inquiries persist regarding various dimensions: causative factors, resultant effects, extent (persistence), critical or crucial developmental windows, and the fundamental biological mechanisms. So far, research efforts have been concentrated on a limited array of fish species. Results are anticipated to exhibit variability among species that differ in their natural habitats, nutritional needs, developmental paradigms, and life history strategies. In future research endeavors, broadening the spectrum of species subjected to programing investigations may yield significant insights into these inquiries. Furthermore, it has been noted that numerous studies document programing effects without longitudinally tracking the entire life course of the organisms involved, as reported by Langley‐Evans [23], a trend that is particularly evident in ichthyological studies, most of which were concluded during the juvenile phase or earlier. Prolonging the extent of experimental investigations, potentially extending across multiple generations [100], may possibly result in substantial benefits for the sectors of biomedicine, environment, and aquaculture.

For the advancement of epigenetic research, it is imperative to differentiate between authentic additive effects and epigenetic influences to enhance the precision of selective breeding aimed at achieving genetic enhancement. Subsequently, there exists a necessity to establish biomarkers for the initial phases of life, which would facilitate the anticipation of adverse outcomes (phenotypes) that may only manifest later in the organism’s life cycle. Epigenetic markers within the genome may serve as valuable biomarkers for this objective. Systematic biomarker evaluations could serve to signal variations in elements triggering nutrition and environment, subsequently impacting fish larvae and the ensuing meat quality in adult fish. This form of proactive monitoring is frequently employed to alert stakeholders regarding the dissemination of diseases related to agriculture. Should nutritional‐driven epigenetic influences prove significant, such monitoring could also contribute to the maintenance of consistent quality in aquaculture products annually, which would subsequently yield substantial implications for the industry, including diminished waste, reduced production of low‐quality products, enhanced consumer confidence, improved export market prospects for salmon products, and increased profitability for producers, processors, and exporters.

Moreover, numerous studies have established that developmental plasticity is indeed present in marine fish, as evidenced by the emergence of a distinct phenotype following an acute challenge several months after the early‐life conditioning event. Nevertheless, as illustrated by their findings, this conditioning may not be enduring and could diminish with time. Consequently, nutritional or metabolic programing methodologies for marine fish larvae ought to consider this likelihood and may benefit from the implementation of regular or short‐term nutritional pulses at intervals during the initial months of the fish’s life to evaluate the attainment of phenotype stabilization.

## Conflicts of Interest

The authors declare no conflicts of interest.

## Author Contributions

Shivendra Kumar was responsible for the conceptualization and writing of the original draft. Aditi Banik and Maneesh Kumar Dubey were responsible for the review and editing of the manuscript. Prem Prakash Srivastava took part in the review and editing. Zsuzsanna J. Sandor was responsible for the funding, writing of the original draft, and review and editing of the manuscript.

## Funding

This work was supported by the Research Excellence Program of the Hungarian University of Agriculture and Life Sciences.

## Data Availability

Data sharing is not applicable to this article, as no new data were created or analyzed in this study.
